# Gender Inequalities in the Health of Immigrants and Workplace Discrimination in Czechia

**DOI:** 10.1155/2014/480425

**Published:** 2014-07-03

**Authors:** Dagmar Dzúrová, Dušan Drbohlav

**Affiliations:** Department of Social Geography and Regional Development, Faculty of Science, Charles University in Prague, Albertov 6, 128 43 Prague 2, Czech Republic

## Abstract

This study analyses the relationship between immigrants' self-reported/rated health (SRH) and their perceived working conditions in Czechia materialized via discrimination, based on the example of Ukrainian immigrants analyzed by gender dimension. The role of age, education, and marital status is also analyzed. A sample of native-born Czechs serves as a reference frame. A cross-sectional design was applied. Using data from two surveys of Ukrainian immigrants in Czechia and a countrywide health interview survey for Czechs, we analyse inequalities in SRH and workplace discrimination loads. Four binary logistic regression models were computed separately for women and men from Ukraine and Czechia to identify the determinants of fair/poor SRH. We found that only Ukrainian immigrant females were heavily exposed to all four measured types of workplace discrimination, thereby modifying and worsening the quality of their SRH. Determinants which are behind respondents' SRH differ between Ukrainian immigrants vis-à-vis Czechs with one exception. The “oldest age group” (41–62) contributes to poorer assessment of SRH among Ukrainian females, Czech females, and Czech males too. The lowest educational level (primary education) correlates with poor SRH within the sample of Czech males.

## 1. Introduction

Health matters associated with international migration in general, and the health of migrants in particular, are crucial public health challenges faced by governments and societies [1]. This notion formed the basis for the resolution on the “health of migrants” which was endorsed by the Sixty-First World Health Assembly [2] in May 2008. On the other hand, information about the health of migrants is rarely available. Obviously, it is difficult to monitor and, consequently, to improve migrants' health.

The most common indicators reflecting a person's health are based on self-rated health (SRH). Knowledge about SRH can help us understand the health status and needs of migrants. For example, in a recently published study by [3], the authors compared SRH among migrant and ethnic minority groups in the EU countries. In harmony with other scientific studies they concluded that most migrants appeared to be disadvantaged as compared to the majority population, even after controlling for age, gender, and socioeconomic factors. As far as regional patterns are concerned, some of the greatest gaps in knowledge are in the former Soviet countries [4].

The globalization of migration flows has increased multicultural diversity, which also importantly contributes to posing “significant challenges for both policy and research” [5]. Regardless of migrant status, international migrants can be at risk of poor physical and mental health and are often excluded from legal frameworks that deal with occupational health and safety [6]. The perceptions of poor health and discrimination are crucial in shaping the migration process. The occupational health of migrants tends to be worse than that of nonmigrants [7]. Migrants are at risk of not receiving the same level of health care due to a combination of several factors including legal status, language, and cultural barriers, whilst occupying low-qualified and high-risk jobs [8].

The nature of the association between health status and work discrimination has repeatedly been demonstrated for various populations [9]. Discrimination is a determinant of an individual's state of health, which is in turn linked with social structure and hierarchy, socioeconomic class, gender, and ethnic group. Analyses show us that for some health outcomes the disparities are worsening over time [10].

International migration movements have increased in size and complexity across most European countries [11–13] and research into the health of migrants is therefore becoming even more important. One of the countries with the fastest growing economic migration (at least until 2008, when the global economic crisis started), especially from so-called third-countries (migrants coming from outside the EU) is Czechia.

The main goal of this paper is therefore to analyse the inequalities relationship between SRH and perceived discrimination in working life based on the example of the immigrant population from Ukraine (see more below). Since migrants' health is one of the most inadequately researched areas from a gender perspective, we will pay special attention to this aspect.

### 1.1. Migration and Related Health Issues in Czechia

Since the Velvet Revolution, a total transformation of the society—from a socialist/communist regime to a democratic, parliamentary system based on a free market economy—has started in Czechia. This transformation process, along with ongoing globalization and the shift towards a postmodern society, has brought significant inflows of immigrants (438,000 foreigners at the end of 2012) [14, 15]. The most numerous immigrant group is Ukrainians (113,000 legally registered migrants in the country as of the end of 2012–15). Most of these migrants came primarily for economic and work-related reasons and they are mostly employed in construction, some industrial sectors, services, or agriculture while taking unskilled, manual, low-paid, so-called “3D” (demanding, dirty, and dangerous) jobs.

Studies have begun to look at various aspects of immigrants' integration in Czechia, albeit focusing predominantly on economic ones [16–18]. In the course of time, some research initiatives also touched on immigrants' health and closely related issues [19–26]. However, research devoted to analysing the role of gender within the migration process is rare in the Czech context; see exceptions to this trend [27, 28].

It seems that topics like differences in the health of migrant populations, in the health care which is being provided, or the working conditions in which migrants work are so far unknown, not well understood, underestimated, or often even ignored. Dealing with migration specifics is one of the challenges for policy and policymakers to balance disparities in labour and social rights. Indeed, all European countries with a substantial percentage of migrants in their population should consider adopting specific migrant health policies [4]. Although the number of insecure jobs has considerably increased over the recent decades [14], as already indicated above, relatively little is known about migrants' health and relationships towards their work and factors that may modify them in Czechia. Accordingly, policymakers are poorly informed about the life style and working conditions in which minorities operate on the Czech labour market. There is no doubt that the continuing lack of data, not to mention comparable data, about these issues makes it difficult for policymakers at national and EU level to efficiently combat the discrimination.

### 1.2. Perceived Discrimination and Health

Generally, current research has looked at the effects of perceived discrimination and confirmed that it is a risk factor for multiple health outcomes [29, 30]. For example, Jasinskaja-Lahti et al. [31] compared perceived discrimination and its influence on psychological stress symptoms and the health status of immigrants in Finland. The results regarding group differences in perceived discrimination were consistent with their previous studies. Perceived discrimination was highly predictive of the psychological well-being and health status of the immigrants (see the EU Anti-Discrimination Directives: (i) Directives 2000/43/EC Racial Equality Directive (racial and ethnic origin for employment, education, social protection and social advantages, goods and services including housing); (ii) Directive 2000/78/EC Employment Equality Directive (age, disability, sexual orientation, religion, or belief in employment)).

Pascoe and Richman [32] made a meta-analytic review of 134 samples and noted that when weighing each study's contribution by sample size, perceived discrimination has a significant negative effect on both mental and physical health. These findings suggest potential pathways linking perceived discrimination to negative health outcomes.

Agudelo-Suárez et al. [30] conducted a survey among 2,434 immigrants in four Spanish cities. 73% of men and 69% of women immigrants reported discrimination due to their immigrant status. Workplace-related discrimination was associated with poor mental health (OR 2.97) and with the worsening of self-rated health over time (OR 2.20). The authors concluded that discrimination may constitute a risk factor for health in immigrant workers in Spain and could explain some health inequalities among immigrant populations in Spanish society.

The health of migrants is becoming very important in Czechia too—a country with very limited experience of dealing with immigrants, in general, and with immigrants' health, in particular.

### 1.3. Hypotheses

Our study is based on testing three basic hypotheses. First, we assume that immigrants who perceive that they are exposed to some type of workplace discrimination/loads would be more likely to report poor health than immigrants not exposed to workplace discrimination. The second hypothesis is that immigrants will differ from the Czech majority population in determinants which modify their poor health. The third hypothesis is based on the thesis that all the above realities will be significantly differentiated by gender dimension.

## 2. Methods

### 2.1. Participants and Study Sample

We applied a quantitative approach using a questionnaire survey as our main research tool. For the purpose of this study we combined two surveys that have recently targeted Ukrainian migrants in Czechia (both were supported by the Czech Science Foundation and we were involved in both (the project “Migration and Development-economic and social impacts of migration upon Czechia as a destination country and Ukraine as a country of origin (with special regard to analysis of remittances),” number P404/10/0581, was finished in 2012 whereas the project: “Migratory patterns of immigrants (and natives) in Czechia: concentration or diffusion processes?” number P404/12/1014, is still ongoing). Both of them, while having different main goals and partly diverse selection criteria, also included some common characteristics and questions related to ascertaining SRH and discrimination issues. Thus, by combining them we gained one robust sample, although at the expense of losing the possibility to include a wide variety of characteristics in our analysis. Furthermore, to be able to better interpret the results, we also work with a reference sample from the Czech population, which provided the information about the same relevant health/discrimination issues.

### 2.2. Questionnaire Surveys and Variables

For the Ukrainian immigrant group, we used results of the following two surveys.

1. In the first questionnaire survey, a total of 321 Ukrainian respondents were successfully contacted between May and October 2012. We excluded subjects with missing data, who were not working or were below 18 or older than 62 years and we analysed data for the remaining 228 respondents. A “snow ball” sampling method was applied. First, when searching for respondents we used our own contacts. Second, we also contacted potential respondents via the established Ukrainian community, namely, their firms, churches, schools, and ethnic associations. Some relevant intergovernmental and nongovernmental organizations were also asked for assistance. The survey was carried out in the capital city of Prague and the Central Bohemia region. Only citizens of Ukraine who had been in Czechia for more than 6 months at the moment of the survey (shorter visits to Ukraine were tolerated but for no longer than 1 month in total) and those who, at the same time remitted money, were qualified to take part in the survey. One family could be represented by only one adult member. In collecting data, there was an attempt to get a heterogeneous group of various types of respondents/migrants mainly in terms of characteristics like sex, age, educational level, and profession.
2. Filling in the questionnaire with the given respondents was arranged and carried out on the spot (“face-to-face” contact; PAPI form—paper and pencil interview) by members of the Czech research team GEOMIGRACE (the team is affiliated with Charles University, Faculty of Science, Department of Social Geography and Regional Development (see: http://www.geomigrace.cz)). Thus, there was a chance to explain anything respondents might not understand while also “checking” their work on the questionnaire. When filling in the questionnaire the respondents could use a Czech, Russian, or Ukrainian version and their work lasted approximately about 45 minutes. Respondents were rewarded for participation.
3. A questionnaire survey of two immigrant groups in Czechia—Ukrainians and Vietnamese—was carried out between March and May 2013 and, in this case, a total of 912 respondents were successfully contacted. In this study, however, we use only the subsample of working Ukrainians, which includes information about 342 Ukrainians aged between 18 and 62 years.
4. For the sampling method, quota sampling was applied with quota characteristics (based on 2011 Census data) represented by sex, age, and region of migrant's residence. Within the sample, immigrants who had already been in Czechia for more than 1 year could participate. Those who had moved at least once inside Czechia were intentionally overrepresented (the main research task of the project was to research internal mobility of immigrants). The data were collected via the interviewers' network of The Centre of Independent Public Opinion Research (under the umbrella of the Institute of Sociology of the Academy of Sciences of the Czech Republic). The aim of the interviewers was to find appropriate respondents (they were supported by information about locations where immigrants may be possibly concentrated), to distribute questionnaires, and then to collect them. The respondents filled in the questionnaire (in self-administered mode) in their mother language and it took on average about 45 minutes. Though the main concern of this research task was primarily concentrated on immigrants' internal mobility and related aspects of their lives, issues relevant to immigrants' health were partly covered too (including SRH and discrimination issues).
5. Data for Czech nationals were extracted from the 2008 European Health Interview Survey (EHIS) carried out by the Institute of Health Information and Statistics of the Czech Republic. From June to October 2008, 1,955 persons aged 15 and over were interviewed from a sample of 3,825 subjects originally selected from the Ministry of Interior Civil Registration Information System, through a two-stage stratified selection, with municipalities being the first stage and respondents the second stage. Data collection was conducted through personal interviews (PAPI form—paper and pencil interview). For the purpose of this study we used data for Czech-born working nationals aged between 18 and 62 (same minimum-maximum as the survey of Ukrainians), including a total of 928 persons.

To sum up, as already mentioned, this study is based on data from three questionnaire surveys. The studied population consisted of 570 immigrants from Ukraine who were living and working (employment and entrepreneurship) in Czechia and of 928 Czechs. The total number of respondents in our sample was 1,498.

The dependent variable, SRH, was measured on a 5-point scale ranging from very good to very poor (the question was “How is your health in general?”) and was categorized as “Good” (good/very good) or “Fair/poor” (fair/poor/very poor).

As for the independent variables, we used sociodemographic and workplace characteristics. Age was categorized into three age groups: 18–30 years, 31–40 years, and 41–62 years. Education was categorized as basic (or less), complete secondary education, and complete university education. In relation to marital status, people were classified as married/cohabitating and other possibilities (single, divorced, or widowed).

Perceived workplace environment was determined by answers to the following four questions. “At your workplace, to what extent are you exposed to

- discrimination (as such)—*D*;
- violence or threat of violence—*V*;
- time pressure or overload of work—*T*;
- risk of accident—*A*”?

Possible answers were not exposed, occasionally/slightly, repeatedly, regularly/strongly, do not know, or refusal and were categorized as “yes, exposed” (if he/she was exposed) or “no exposure.”

### 2.3. Statistical Analysis

The data were organized into a database and further analyzed using SPSS 18 software. The basic descriptive statistics were obtained. We analysed sociodemographic and workplace variables in the four subgroups (Czech males and females and Ukrainian males and females). In the next steps of the analysis, binary logistic regression was applied. Multivariate odds ratios (OR) for fair/poor SRH (1—fair/poor health and 0—good health) were calculated and 4 logistic regression models fitted separately for women and men from Ukraine and Czechia to identify the determinants of fair/poor SRH. We added three sociodemographic characteristics (age, education level, and marital status) and one summarizing workplace environment coded 0–4. 0 indicates that the individual was not exposed to any workplace discrimination load (“partial discrimination”); the value 4 is loaded with all four selected workplace discrimination loads.

To sum up, to our knowledge, this is one of the first studies with sufficient data (a more robust sample, albeit not gained through a probability sampling method) to investigate the effects of work discrimination and other selected sociodemographic characteristics on the SRH of immigrants, specifically, immigrants from Ukraine. The disadvantage of this novelty is that there are not as yet any similar results in Czechia that we can refer to or we can compare with.

## 3. Results

A total of 1,498 working individuals (age 18–62 years) were analyzed in the study. Out of this sample, 38.1% of respondents came from Ukraine and 61.9% from Czechia.  Table 1 provides a description of the sample according to the prevalence rate of fair/poor rated-health disaggregated by age groups, education level, marital status, and four types of “overall workplace discrimination” (discrimination as such, violence or threat of violence, time pressure or overload of work, and risk of accident). The most significant differences in workplace discrimination between Ukrainians and Czechs were found in perceived workplace discrimination as such (males: Ukrainians 29% versus Czechs 4%; females: Ukrainians 38% versus Czechs 7%).

The second part of Table 1 provides a description of the prevalence rate of fair/poor SRH. Poor SRH was reported by 23.6% of the sample, whilst females from Ukraine declared the poorest health (28.5%). Results confirmed that poor health is common among older adults and the values increase with age in all four samples. Educational level was inversely related to SRH among immigrant males, but not among immigrant females, where those with university education had the worst SRH. Rather bad SRH was higher in the group of married people than for others (single, widowed or divorced). The highest prevalence of poor health was among people exposed to workplace discrimination (32.6%), especially among Ukrainian females. However, these data have to be interpreted carefully since they spring from different structures of the sample populations and also vary in absolute terms.

Table 2 shows the structure of the four types of workplace (*D*, *V*, *T*, and *A*) exposure for 4 compared samples (males versus females and Ukrainians versus Czechs). The first part of the table (by rows) informs us about combinations of individual work place expositions. Altogether, we found 15 combinations. In the total sample, one quarter (25.7%) did not report any workplace discrimination loads. On the other hand, the most frequent exposition/load is a combination of (1) time pressure/overload of work and (2) risk of accident (Exp. *TA* = 26.7%). The time pressure alone was detected as the second most important exposition/load (Exp. *T* = 19.9%) followed by the accident (Exp. *A* = 10.4). Importance of all other combinations was more or less negligible.

As for the males, the most frequent exposition/load is time pressure in combination with the risk of accident (Exp. *TA*). As far as Ukrainian males are concerned, it is 18.5%, as for Czech males, it is even 43.2%. When analyzing females, the most frequent is time pressure (Exp. *T*) exposition/load. Regarding Ukrainian females it is 20.3% whereas concerning Czech females it reaches 25.9%. The second part of the table brings numbers of respondents' simultaneous exposures ranging from 1 to 4. In the total sample, 32.2% reported one load, 31.5% two loads, and 7.9% three loads and 2.8% were exposed to four discrimination loads at their workplace simultaneously. The exposition to two factors is the most typical of the males. As for Ukrainian males it is 24.9% and regarding Czech males it is 44.4%. Females, as compared to males, are the most frequently exposed to only one factor (Ukrainian females 27.5%, Czech females 38.9%). In fact, these results correspond to the findings we have presented above.

Table 3 demonstrates the four models of associations between fair/poor SRH and self-reported workplace discrimination loads and sociodemographic variables (for Ukrainian and Czech males and females). The number of workplace discrimination loads (OR = 4.05, 95% CI = [1.25–13.08] for those with one load; OR = 4.89, 95% CI = [1.47–16.30] for those with two loads; OR = 13.18, 95% CI = [3.49–49.81] for those with three loads; and OR = 6.58, 95% CI = [1.18–36.82] for those with four loads when compared with those without any discrimination load and age (OR = 6.36, 95% CI = [2.21–18.30]) for those in the 41–62 year age group when compared with individuals aged 18–30 years were identified as significant predictors of SRH for Ukrainian immigrant females. On the other hand, there was no significant relevant factor (important predictor of SRH) found among Ukrainian males; no statistical correlation between poor health and given independent variables was identified. Furthermore, age was also detected as a relevant factor related to SRH in the case of both Czech males and Czech females in the sense that the higher the age the worse the declared SRH, though this relationship is less intensive than that for Ukrainian females (see particular parameters in Table 3).

### 3.1. Limitations

This study has some limitations that are worth mentioning.

- The two surveys this analysis is based on originated in different time periods (May–October 2012 and March–May 2013) and regions. Whereas the first investigation was carried out in the capital city of Prague and the Central Bohemia region, the second was done throughout whole Czechia. The data samples are slightly different also in regard to employment and educational characteristics (due to regionally specific work opportunities). The different structures of the respondents have logically had some impact on differences in the crude values of the dependent variable, SRH.
- There are differences between immigrant and Czech data samples, too. Males dominated in the group of the immigrants (again logical in the context of labour migration). On the other hand, in the Czech population sample, gender characteristics are more or less balanced. We are aware of the differences in the structures described above and therefore used methods of logistic regression which makes it possible to overcome problems with different structures entering the analyses. Obviously, the immigrant data are unique and variation between them is not significant in terms of our goals.
- All the data were self-reported. Hence, despite care taken with methodology, self-reporting may partly be influenced by memory and/or social desirability factors.

## 4. Discussion and Conclusions

Several aspects are worth discussing. It does not seem that the “healthy immigrant effect” is recognised in many developed immigration countries [33, 34] can be proved through our results in Czechia. Differences between SRH as they are declared by Ukrainian immigrants vis-à-vis native-born Czech population are overall not big. This does not mean, however, that these two groups do not differ significantly when comparing some partial elements. Overall differences in SRH between gender categories are more apparent in favour of males. Hence, in harmony with many other studies [33, 34], we also confirmed that position of immigrant females (vis-à-vis immigrant males) in the host Czech society and its labour market is difficult and vulnerable to many possible forms of discrimination and threats. There are two important aspects that may contribute significantly to this fact. First, whereas males can fulfil their main “breadwinner position” through finding a job and working hard, females' role is often also oriented towards other tasks and interests. It is usually important for females to maintain and further develop their “social structures” and ties, be it within the family or broader social units. This requirement is difficult to meet abroad since a part of a family, including their child or children, often remains at home in Ukraine (almost half of the Ukrainian female respondents were married) and due to working hard immigrant females have no time to socialize either with their compatriots or with the Czech majority. Moreover, the poorest health (SRH) found for highly educated female immigrants may indicate their frustration when their job/work in Czechia (as in many other immigration countries) is often manually, not intellectually demanding, and does not correspond at all to their education (“prestige”) and, indeed, expectations. They feel their human capital is lost. This may contribute to the worsening of their SRH (for a similar effect see [16]). In fact, our conclusions correspond to Lynam's [35] comment that…“manual immigrant women are exposed to the cumulative burden of socio-economic and gender-related disadvantages and disempowerments, together with their experience of marginalization” [35].

We tested three hypotheses. All were, albeit not fully, confirmed.

First, among the studied samples (Ukrainian males and females and Czech males and females), only Ukrainian immigrant females were heavily exposed to all four measured types of workplace discrimination loads within the given model, while also being more likely to report rather poor health. Within other analysed samples respondents' possible workplace discrimination did not modify the quality of their SRH. This confirms how difficult the situation of immigrant females in the labour market of a host country often is (they are predisposed to various forms of discrimination; they are more prone to various threats); Czechia as the immigration/host country is no exception to this trend.

The second tested hypothesis holds true in the sense that determinants (which were used within the analysis through binary logistic regression) which are behind respondents' quality of health are different among Ukrainian immigrants vis-à-vis Czechs with one exception. The “oldest age” (41–62) contributes jointly to poorer assessment of SRH among Ukrainian females and Czech females, and indeed Czech males too. Workplace discrimination in its four different forms is clearly tied to poorer SRH but only in the case of Ukrainian females. On the other hand, it seems that the lowest educational level (primary education) correlates with poor SRH within the sample of Czech males.

On one hand, the results show us how complicated relationships between SRH and workplace discrimination and also the given sociodemographic characteristics are. On the other hand, the results clearly call for policy interventions to more effectively prevent workplace discrimination (as a whole and in its various forms) in the Czech labour market targeted especially towards Ukrainian immigrants, in general, and Ukrainian immigrant females, in particular (as the results from testing the third hypothesis tell us).

Further studies with a larger sample size are needed to further explore and test our findings. Moreover, in addition to the quantitative approach, a qualitative one is also necessary in order to understand the issue better and to gain in-depth knowledge and nuances. Czech migration and health policy should focus not only on supporting foreigners' integration into society but also especially on the prevention of possible problems within immigration communities. The primary goal should be to ensure the same conditions of life as the majority society.

The health of immigrants is a topical issue and it calls for further studies tackling various aspects in this field. The current situation resembles “the precarious safety of an ice float.”

## Figures and Tables

**Table 1:** Sample characteristics, workplace exposition, and prevalence of fair/poor self-rated health.

	Study sample										Prevalence of fair/poor health									
	Males				Females				Total		Males				Females				Total	
	Ukrainian		Czech		Ukrainian		Czech				Ukrainian		Czech		Ukrainian		Czech			
	*N*	%	*N*	%	*N*	%	*N*	%	*N*	%	*N*	%	*N*	%	*N*	%	*N*	%	*N*	%
Sociodemographic characteristics																				
Age groups																				
18–30	72	19,6	125	24,0	59	29,2	88	21,6	344	23,0	12	16,9	15	12,0	8	13,8	12	13,6	47	13,7
31–40	130	35,3	160	30,7	63	31,2	120	29,5	473	31,7	24	18,5	26	16,3	13	20,6	24	20,0	87	18,4
41–62	162	44,0	236	45,3	79	39,1	199	48,9	676	45,3	40	24,7	72	30,5	36	46,2	70	35,2	218	32,3
Education level																				
Primary	174	47,3	263	50,5	60	29,7	160	39,3	657	44,9	44	25,4	70	26,6	17	28,3	50	31,3	181	27,6
Secondary	136	37,0	187	35,9	81	40,1	194	47,7	598	40,8	22	16,2	31	16,6	19	23,5	47	24,2	119	19,9
University	54	14,7	58	11,1	57	28,2	40	9,8	209	14,3	9	16,7	9	15,5	20	36,4	7	17,5	45	21,7
Marital status																				
Married	221	60,1	308	59,1	94	46,5	252	61,9	875	58,5	49	22,3	76	24,7	31	33,0	70	27,8	226	25,9
Other	147	39,9	213	40,9	108	53,5	154	37,8	622	41,5	27	18,4	37	17,4	26	24,5	36	23,4	126	20,3

Exposure at the workplace																				
Discriminations																				
Not exp.	242	65,8	487	93,5	118	58,4	364	89,4	1 211	83,9	46	19,1	100	20,5	25	21,6	92	25,3	263	21,8
Exposed	106	28,8	21	4,0	77	38,1	29	7,1	233	16,1	26	24,5	10	47,6	29	37,7	11	37,9	76	32,6
Violence or threat of violence																				
Not exp.	304	82,6	491	94,2	166	82,2	385	94,6	1 346	93,7	60	19,8	105	21,4	41	25,0	100	29,4	306	22,8
Exposed	44	12,0	17	3,3	21	10,4	8	2,0	90	6,3	11	25,0	5	26,0	8	38,1	4	50,0	28	31,1
Time pressure or overload of work																				
Not exp.	137	37,2	167	32,1	79	39,1	173	42,5	556	38,5	22	16,2	39	23,4	10	13,0	38	22,0	109	19,7
Exposed	211	57,3	343	65,8	116	57,4	219	53,8	889	61,5	50	23,7	70	20,4	43	37,1	64	29,2	227	25,5
Risk of accident																				
Not exp.	191	51,9	172	33,0	142	70,3	243	59,7	748	52,3	34	17,9	42	24,4	31	22,1	60	24,7	167	22,4
Exposed	150	40,8	336	64,5	47	23,3	149	36,6	682	47,7	35	23,3	67	19,9	19	40,4	44	29,5	165	24,2

Total∗	368	100	521	100	202	100	407	100	1 498	100	76	20,7	113	21,7	57	28,5	106	26,0	352	23,6

*Note.* ∗The difference between the total and the sum of the individual categories-missing values.

**Table 2:** Structure of the four types of workplace exposures: Ukrainian and Czech males and females.

*D*: discriminations										
*V*: violence	Males				Females				Total	
*T*: time pressure	Ukrainian	Czech	Ukrainian	Czech	Ukrainian	Czech	Ukrainian	Czech		
*A*: accident	*N*	*N*	%	%	*N*	*N*	%	%	*N*	%
Without exp.	103	76	31,3	15,1	59	121	32,4	31,3	359	25,7
Exp. *D*	10		3,0		12	2	6,6	,5	24	1,7
Exp. *DV*	1		,3		3		1,6		4	0,3
Exp. *DT*	13	2	4,0	,4	21	13	11,5	3,4	49	3,5
Exp. *DA*	4	3	1,2	,6					7	0,5
Exp. *DV* *T*	4	1	1,2	,2	2	1	1,1	,3	8	0,6
Exp. *DV* *A*	3		,9		1		,5		4	0,3
Exp. *D* *TA*	37	10	11,2	2,0	20	13	11,0	3,4	80	5,7
Exp. *DV* *TA*	24	5	7,3	1,0	10		5,5		39	2,8
Exp. *V*					1	1	,5	,3	2	0,1
Exp. *VT*	3		,9		2	1	1,1	,3	6	0,4
Exp. *VA*		1		,2					1	0,1
Exp. *V* *TA*	2	10	,6	2,0	2	4	1,1	1,0	18	1,3
Exp. *T*	52	90	15,8	17,9	37	100	20,3	25,9	279	19,9
Exp. *A*	12	87	3,6	17,3		47		12,2	146	10,4
Exp. *TA*	61	217	18,5	43,2	12	83	6,6	21,5	373	26,7

Without exp.	103	76	31,3	15,1	59	121	32,4	31,3	359	25,7
Exposed by 1 factor	74	177	22,5	35,3	50	150	27,5	38,9	451	32,2
Exposed by 2 factors	82	223	24,9	44,4	38	97	20,9	25,1	440	31,5
Exposed by 3 factors	46	21	14,0	4,2	25	18	13,7	4,7	110	7,9
Exposed by 4 factors	24	5	7,3	1,0	10		5,5		39	2,8

Total valid	329	502	100,0	100,0	182	386	100,0	100,0	1 399	100,0

Missing	39	19			20	21			99	
Total	**368**	**521**	** **	** **	**202**	**407**	** **	** **	**1 498**	** **

**Table 3:** The association of fair/poor self-rated health with self-reported workplace loads and demographic variables: Ukrainian and Czech males and females (Adj. odds ratio (OR) and 95% CI, 4 log. models).

	Adj. OR	95% CI		Adj. OR	95% CI		Adj. OR	95% CI		Adj. OR	95% CI	
	Ukrainian males			Ukrainian females			Czech males			Czech females		
Age groups												
18–30 (ref.)	1			1			1			1		
31–40	,985	,408	2,379	,902	,274	2,967	1,578	,717	3,473	1,224	,524	2,859
41–62	1,779	,769	4,116	**6,361**	**2,211**	**18,298**	**3,508**	**1,677**	**7,338**	**3,106**	**1,434**	**6,728**
Education level												
Univ. (ref.)	1			1			1			1		
Primary	2,065	,800	5,327	,514	,182	1,451	**2,318**	**1,010**	**5,321**	1,562	,573	4,256
Secondary	1,156	,423	3,164	,425	,159	1,133	1,264	,527	3,030	1,297	,482	3,485
Marital status												
Other (ref.)	1			1			1			1		
Married	1,071	,573	2,004	1,457	,650	3,265	1,144	,678	1,931	,957	,559	1,639

Workplace discrimination by factors												
Not exposed (ref.)	1			1			1			1		
Exposed by 1 factor	1,313	,582	2,958	**4,050**	**1,254**	**13,082**	,665	,341	1,295			
Exposed by 2 factors	1,780	,830	3,816	**4,891**	**1,468**	**16,297**	,578	,302	1,108	1,073	,588	1,958
Exposed by 3 factors	2,140	,904	5,068	**13,180**	**3,487**	**49,814**	,818	,266	2,517	1,879	,999	3,534
Exposed by 4 factors	1,277	,370	4,416	**6,578**	**1,175**	**36,821**	3,041	,439	21,074	2,687	,890	8,118

Educational level was confirmed as a significant determinant of poor health (SRH) but only in the case of Czech males (OR = 2.32, 95% CI = [1.01–5.32]—the lower the education the worse the parameters of SRH (taking university education as the reference frame)).

Marital status was not significantly associated with SRH in any analysed samples.
